# Vaginal flora during pregnancy and subsequent risk of preterm birth or prelabor rupture of membranes: a nested case–control study from China

**DOI:** 10.1186/s12884-023-05564-y

**Published:** 2023-04-12

**Authors:** Xiaomei Liu, Shuting Si, Li Huang, Meiliang Zhang, Wenya Chen, Liquan Wang, Yunxian Yu

**Affiliations:** 1 Department of Gynecology and Obstetrics, Yiwu Maternal and Children Hospital, Yiwu, China; 2 Department of Science and Education, Yiwu Maternal and Children Hospital, Yiwu, China; 3 Department of Epidemiology & Health Statistics, School of Public Health, School of Medicine, Zhejiang University, Hangzhou, China; 4 Department of Public Health, and Department of Anesthesiology, The Second Affiliated Hospital, School of Medicine, Zhejiang University, Hangzhou, China; 5 Department of Obstetrics, The Second Affiliated Hospital, School of Medicine, Zhejiang University, Hangzhou, Zhejiang China

**Keywords:** Vaginal flora, Preterm birth, Prelabor rupture of membranes

## Abstract

**Background:**

The findings of the association of vaginal flora with preterm birth (PTB) or prelabor rupture of membranes (PROM) were conflicts. Moreover, vaginal flora was different by ethnicity and the evidence from China was limited.

**Methods:**

This study was a nested case control study, based on Yiwu birth cohort. We assessed vaginal microbiota in the second or third trimester, using 16S rDNA Amplicon Sequencing and explored the association between the diversity and composition of vaginal flora and PTB or PROM.

**Results:**

We finally included 144 pregnant women. In present study, the alpha diversity of TPROM (Term prelabor rupture of membranes) samples was lower than that of full term samples (Chao1 index: *P* < 0.05). When we further categorized PTB (Preterm birth) into SPB (PTB without PROM) and PPROM (Preterm prelabor rupture of membranes), there was no difference between SPB and full term. In addition, we found that the proportion of PCoA2 in TPROM group was different from that in full term group and preterm group. The difference between groups was significant according to anosim analysis (*R* = 0.059, *P* < 0.001). With LEfSe (Linear discriminant analysis Effect Size) analysis, we found that the abundance of *Lactobacillus* in the vaginal flora of pregnant women with preterm birth was the highest (*P* = 0.003).

**Conclusion:**

In Chinese pregnant women, the alpha diversity in TPROM group was significantly lower than that in both PTB and full term group. However, there was no difference between PTB and full term. *Lactobacillus* was the most abundant in preterm birth group. More studies should be conducted to confirm our findings.

**Supplementary Information:**

The online version contains supplementary material available at 10.1186/s12884-023-05564-y.

## Introduction

Preterm birth (PTB) is defined as birth delivery at less than 37 gestational weeks. In 2019, WHO reported that the incidence of preterm birth in China was 6.9% [1]. Preterm birth and its related complications are the leading cause of death in children under five years old worldwide [2] and survivors of PTB are still associated with an increased risk for long-term neurological dysfunction, chronic lung disease, retinopathy, behavioral disorders and learning difficulties [3, 4]. Prelabor rupture of membranes (PROM) is defined as rupture of membranes before the commencement of labor. PROM occurs among the birth deliveried before and after 37 weeks of gestation is referred as Preterm prelabor rupture of membranes (PPROM) and Term prelabor rupture of membranes (TPROM), respectively [5]. The reported incidence of TPROM and PPROM in China ranged from 12.1% to 15.6% and 2.0% to 3.1%, respectively [6–8]. Those with PROM, including TPROM and PPROM, had higher risk for a number of adverse outcomes, including intrauterine infection, postpartum hemorrhage, fetal distress, even long-term adverse outcomes [9, 10]. However, the clinical evidence quality for PROM management is still low, it is still a worthy topic to predict the risk of PROM and further explore the prevention of PROM [11]. The etiology of PTB and PROM has been shown to be complicated. The vaginal microbiome during pregnancy acts as a barrier to bacteria and pathogens and has been linked to preterm birth and other adverse perinatal outcomes [12–15]. A nested case–control study from UK tertiary referral hospital found that vaginal bacterial load was associated with early sPTB/PPROM recurrence[16]. Edward et al. [17] found that abnormal vaginal flora was associated with intrauterine infection, and retrograde infection of pathogenic microorganisms in the reproductive tract was the main cause of PPROM. The absence of *Lactobacillus spp.* and polymicrobial colonization of the vagina has been recognized as risk factors for PPROM [18]. However, Roberto et al. and Young-Ah et al. found no significant association between any particular community type or microbial taxa and preterm birth [19, 20]. The inconsistent results could be partly due to different study designs, study populations, gestational age at sampling and bioinformatics and statistical analysis methods. Ethnicity plays an important role in PTB, PROM and Vaginal flora [21–23]. However, to our knowledge, no studies from China explored the association of vaginal flora with PTB and PROM by 16S rDNA sequencing.

Therefore, we conducted a nested case–control study in Zhejiang Yiwu to explore the association of maternal pregnancy vaginal flora diversity and composition, determined using 16S rDNA sequence-based methods, with PTB and PROM.

## Methods

### Study design and population

Yiwu city is located in the middle of Zhejiang Province, China, east longitude 119°49 ' ~ 120°17', north latitude 29°02 ′13 " ~ 29°33′ 40", the population size is 1,859,390 residents. Yiwu Maternal and Child Care Hospital is a tertiary hospital with obstetrics, gynecology and pediatrics as its specialty and integrating medical care, prevention, teaching and scientific research. The amount of annual delivery in this hospital is more than 10,000 cases. Yiwu Birth Cohort (YBC) is a running prospective cohort study conducted in Yiwu Maternal and Child Care Hospital, since January 2018. Pregnant women were recruited at their first prenatal visit (8 to 12 gestational weeks). Inclusion criteria: (1) agreed to participate in the cohort and provided informed consent; (2) would accomplish perinatal examination and deliver birth in Yiwu Maternal and Children Hospital; (3) aged from 18 to 45 years old. Exclusion criteria: (1) serious chronic or acute disease history; (2) any mental disorder before pregnancy; (3) any threatened abortion or embryonic dysplasia; (5) unable to complete the questionnaire due to intellectual issues. Meanwhile, the participants from YBC met the following criteria were included in this manuscript. Inclusion criteria: (1) the biological specimen of vaginal discharge was available. Exclusion criteria: (1) with syphilis, HIV and other reproductive tract transmits diseases; (2) antibiotics or antifungal drugs were administered before sampling within 30 days. Due to February 2021, 2980 pregnant women accepted to participant in the YBC and 848 participants had biological specimen of vaginal discharge. And there was no difference about maternal age and parity between groups (Supplementary Table 1). Among those with viginal sample, 48 cases were preterm birth. Finally, the nested case–control study design was conducted in this manuscript. The mothers with preterm birth, full term without PROM, and TPROM were matched 1:1:1 by parity, age within 5 years, sampling gestational age within 2 weeks. The study protocol was approved by the ethics board of Yiwu Maternal and Child Care Hospital.

### Clinical data collection

We extracted maternal age, parity, delivery gestational age, maternal last menstrual period from electronic medical records. In addition, we also extracted the medication history of antibiotics or antifungal drugs and the information of pregnancy complications, including gestational diabetes, pregnancy induce hypertension, amniotic fluid anomaly (including hydramnios and oligohydramnios), infectious diseases, placenta previa and preeclampsia. Gestational age at delivery was assessed by maternal last menstrual period (LMP) in combination with ultrasound assessments. We defined PTB as gestational age at delivery of < 37 week. Prelabor rupture of membranes (PROM) was defined as the outflow of amniotic fluid from around the fetus before the onset of uterine contractions, according to the American College of Obstetricians and Gynecologists Practice Bulletin (American College of Obstetricians and Gynecologists, 2020) and were categorized into term PROM (TPROM) and premature PROM (PPROM) [5, 24]. SPB was defined as preterm birth without PROM.

### Vaginal sample collection

Vaginal swab was collected by trained research staff. During routine physical examinations of pregnancy at the second or third trimester (The mean collected gestational age was 26 weeks), the vagina was opened with a speculum, vaginal swabs were collected from the cervix and posterior fornix with a sterile cotton ball, and the cotton ball was placed into a tube preloaded with 1 ml sterile phosphate buffer (PBS) and immediately stored at -80 °C until assayed.

### Extraction of genome DNA

Total genomic DNA from samples was extracted using SDS method. DNA concentration was determined by Nanodrop. The purity and integrity could be evaluated through the 1% agarose gel electrophoresis.

### 16S rRNA gene sequencing and sequence data processing

According to the concentration, DNA was diluted to 1 ng/μl using sterile water. 16S rDNA was amplified used the specific primer with the barcode. Sequencing libraries were generated using TIANSeq Fast DNA Library Prep Kit (illumina) (TIANGEN Biotech). The library quality was assessed on the Qubit@ 2.0 Fluorometer (Thermo Scientific) and Agilent Bioanalyzer 2100 system. At last, the library was sequenced on the Illumina platform using the 2 × 250 bp paired-end protocol.

### Statistical analysis

On average, 93,530 records of original data were measured for each sample, 89,888 records of effective data were obtained after filtered, denoised, spliced and removed of chimerism, with an effective rate of 96.11%. After that, it would run the de-duplication operation on the obtained valid data to obtain the de-duplication sequence ASV (Amplicon sequence variant). Then, we removed the Singletons ASVs (ASVs with only 1 sequence total in the entire sample). Finally, in order to ensure comparability of species diversity between samples, the diversity core-metrics-phylogenetic command in QIIME2 software was used for standardization, and the standardized data depth was set to 95% of the minimum sample sequence amount. The normalized sample sequencing depth was 43,694, and the number of ASV was 6,060. Species annotation was performed using QIIME2 software. For 16S, the annotation database is Silva Database. In order to study phylogenetic relationship of each ASV and the differences of the dominant species among different samples (groups), multiple sequence alignment was performed.

Alpha diversity was applied in analyzing complexity of species diversity for a sample [25]. Beta diversity analysis was used to evaluate differences of samples in species complexity. Principal Coordinate Analysis (PCoA) was performed to obtain principal coordinates and visualize differences of samples in complex multi-dimensional data. A matrix of weighted or unweighted unifrac distances among samples obtained previously was transformed into a new set of orthogonal axes, where the maximum variation factor was demonstrated by the first principal coordinate, and the second maximum variation factor was demonstrated by the second principal coordinate, and so on. To confirm differences in the abundances of individual taxonomy or function annotation among PTB, Term and PPROM groups, Metastats and STAMP software was utilized. LEfSe analysis (Linear discriminant analysis Effect size, LDA score threshold: 4) was used for the quantitative analysis of biomarkers within different groups. Kruskal–Wallis test was used to analyze the difference of diversity among PTB, full term and TPROM groups. Analysis of similarities (Anosim) was conducted to test whether differences between groups are significantly greater than differences within groups. When comparing the characteristic among preterm, term and TPROM, One-way Analysis of Variance and Chi square test were used for continuous variables and categorical variables, respectively. All analyses, except where specifically noted, were performed in R (www.r-project.org). *P* value less than 0.05 was considered statistically significant.

## Results

### Characteristics of the study population

As shown in Table 1, there was no significant difference among preterm cases (*n* = 48; mean delivery week: 34.83 ± 1.55), TPROM group (*n* = 48; mean delivery week: 39.50 ± 0.68) and full term group (*n* = 48; mean delivery week: 38.88 ± 0.70) in gestational week at sample collection, maternal age, parity and pregnancy complications (including gestational diabetes, pregnancy induced hypertension, Amniotic fluid anomaly, hepatitis B virus infection, placenta previa and preeclampsia).

**Table 1:** Comparisons of characteristic among preterm, term and TPROM

Variables	Preterm (*N* = 48)	Term (*N* = 48)	TPROM (*N* = 48)	*P*
Delivery gestational age, week	34.83 ± 1.55	39.50 ± 0.68	38.88 ± 0.70	< 0.001
Gestational week at detection, week	26.12 ± 5.95	26.29 ± 6.02	26.00 ± 6.32	0.973
Maternal age, years	28.58 ± 5.34	28.69 ± 4.90	29.15 ± 4.48	0.837
Parity				0.495
0	22 (45.8)	22 (45.8)	22 (45.8)	
1	21 (43.8)	21 (43.8)	25 (52.1)	
≥ 2	5 (10.4)	5 (10.4)	1 (2.1)	
GDM				0.508*
No	43 (89.6)	46 (95.8)	46 (95.8)	
Yes	5 (10.4)	2 (4.2)	2 (4.2)	
PIH				0.329*
No	46 (95.8)	48 (100.0)	48 (100.0)	
Yes	2 (4.2)	0 (0.0)	0 (0.0)	
Amniotic fluid anomaly				1.000*
No	46 (95.8)	46 (95.8)	46 (95.8)	
Yes	2 (4.2)	2 (4.2)	2 (4.2)	
HBV infection				0.909*
No	46 (95.8)	45 (93.8)	44 (91.7)	
Yes	2 (4.2)	3 (6.2)	4 (8.3)	
Placenta previa				0.329*
No	46 (95.8)	48 (100.0)	48 (100.0)	
Yes	2 (4.2)	0 (0.0)	0 (0.0)	
Preeclampsia				1.000*
No	47 (97.9)	48 (100.0)	48 (100.0)	
Yes	1 (2.1)	0 (0.0)	0 (0.0)	

*TPROM* Premature rupture of membranes at term, *GDM* gestational diabetes, *PIH* pregnancy induce hypertension, *HBV* Hepatitis B virus

^*^Fisher exact test

Based on the rarefaction curve, the sequencing depth was sufficient to describe the diversity of the samples (Supplementary Fig. 1a and b).

### Vaginal flora alpha diversity was associated with PTB or PROM

**Fig. 1:**
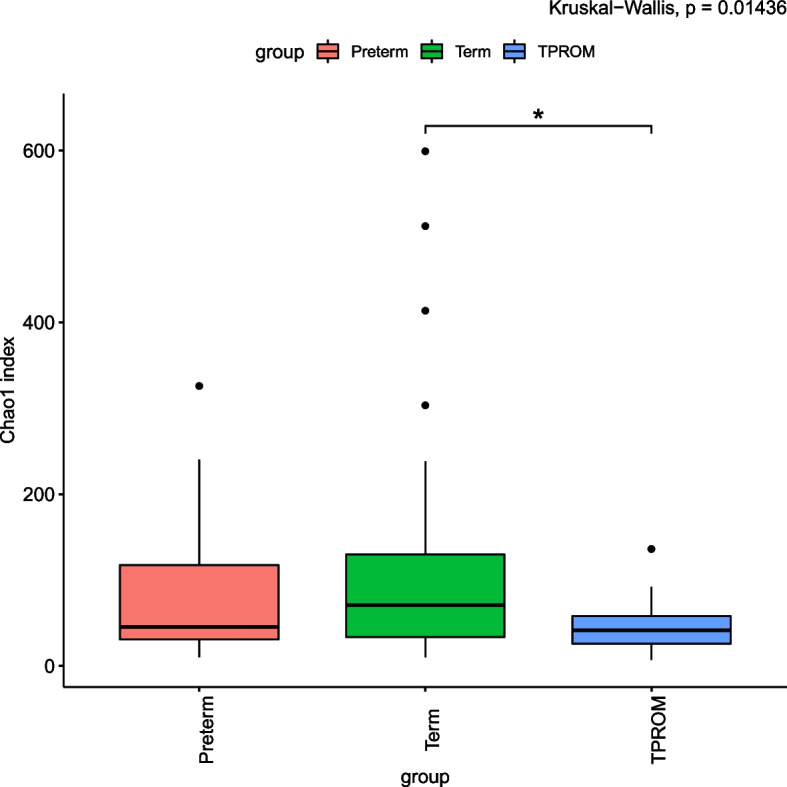
The comparison of alpha diversity (Chao 1 index) among PTB, Term and TPROM

The abundance and diversity of vaginal flora were significantly different among preterm, full term and TPROM group (Chao1 index: *P* = 0.014). And the alpha diversity in mothers with TPROM was lower than that in the mothers with full term (Chao1 index: *P* < 0.05, Fig. 1). When we further categorized preterm birth into SPB and PPROM, there was no difference between SPB and full term. However, the alpha diversity in TPROM group were lower than that in both SPB and full term group (Chao1 index: *P* < 0.05, Supplementary Fig. 2a). In addition, the alpha diversity of PPROM was lower than that of SPB and full term, although it was not statistically significant (Supplementary Fig. 2b and c).

**Fig. 2:**
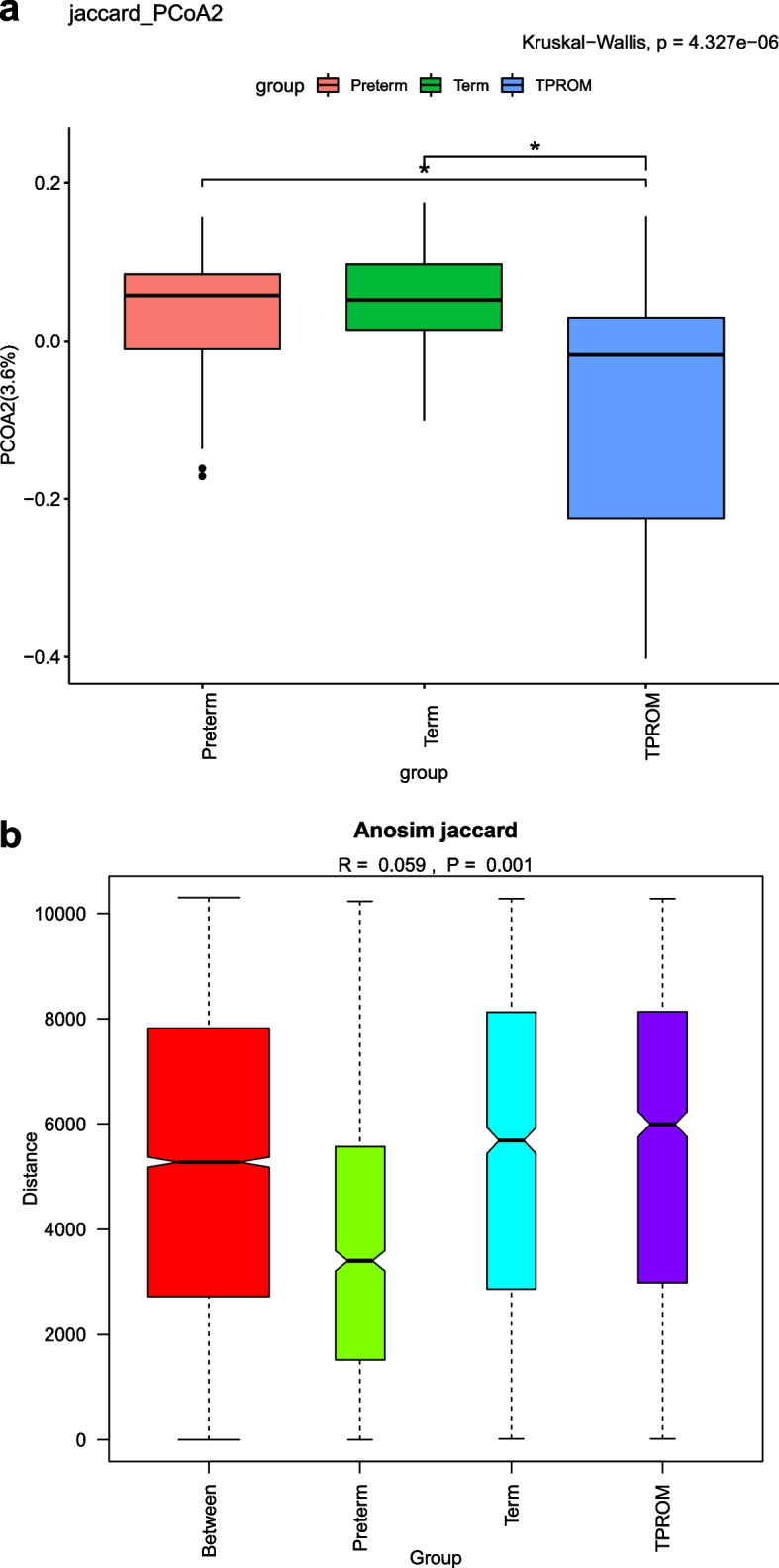
**a** The Principal coordinates analysis among PTB, Term and TPROM groups. **b** The analysis of similarities among PTB, Term and TPROM groups

### Vaginal flora beta diversity was associated with PTB or PROM

Beta diversity estimates the biodiversity between samples. We conducted PCoA and anosim analysis, using jaccard distance to detect the beta diversity among groups. We found that the proportion of PCoA2 in TPROM group was different from that in full term group and preterm group and the difference between groups was significant, according to anosim analysis (R = 0.059, *P* < 0.001, Fig. 2a and b). The results were similar when only kept SPB and the corresponding controls (*R* = 0.014, *P* < 0.001, Supplementary Fig. 3a and b). However, probably due to the limited sample size of PPROM, we failed to find the significant difference when only compared PPROM with the corresponding controls (Term and TPROM) (*R* = 0.019, *P* = 0.177, Supplementary Fig. 3c and d). When compared the beta diversity between SPB group and PPROM group, no statistically significant difference was found (*R* = 0.033, *P* = 0.226, Supplementary Fig. 3e and f).

### Vaginal flora relative abundance composition was associated with PTB or PROM

**Fig. 3:**
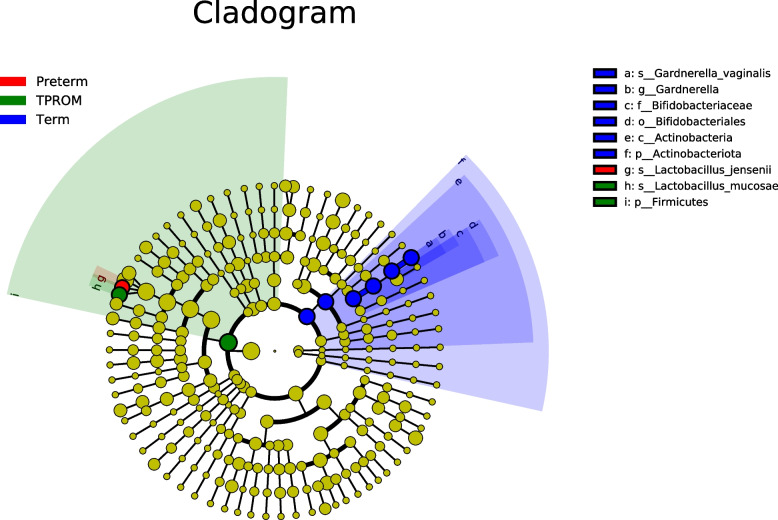
Cladogram describing differentially abundant vaginal microbial observed between women subsequently experiencing PTB, Term and PPROM as identified using LEfSe analysis

By comparing the ASV/OTU (operational taxonomic units) representative sequence with the microbial reference database, the species classification information corresponding to each ASV/OTU were be obtained, and then the community composition of each sample at each level (phylum, class, Order, family, genus, species) were be counted. The distribution of vaginal flora relative abundance composition among PTB, full term and TPROM were presented for taxonomy. To identify the vaginal flora biomarkers during pregnancy associated with the risk of preterm birth and PROM, we used LEfSe on 16S rRNA data. The abundance of *Lactobacillus_jensenii* in the vaginal flora of pregnant women with preterm birth was the highest (*P* = 0.003). Samples from pregnant women with term birth were enriched in bacteria phylum Actinobacteria while samples from pregnant women with TPROM were enriched in bacteria Firmicutes (Fig. 3).

## Discussion

In current study, we found that the abundance and diversity of vaginal flora were significantly different among PTB, full term and TPROM group. The alpha diversity of vaginal flora among participants with TPROM were significantly lower than that in both PTB and full term group. However, there was no difference between PTB and full term. In terms of beta diversity, TPROM group was different from that in Term group and preterm group. Based on the LEfSe analysis, *Lactobacillus* was the most abundant in preterm birth group.

The composition of the vaginal flora was different among Asian, Caucasian, Black and Hispanic pregnant women and Asian and Caucasian had the lowest alpha diversity [23].

Study from South Korea and London, using a 16S metagenomics approach, showed that pregnant women at risk for PPROM had greater abundance and diversity at the time of admission than those tended to preterm birth without PROM, which indicated the importance of vaginal flora abundance and diversity on PROM [20, 26]. However, in terms of PPROM and preterm birth without PROM, we found the opposite trend, though not yet statistically significant. Moreover, when compared to pregnant women subsequently delivered to term, those with TPROM also had lower alpha diversity. There were few studies focused on TPROM and PPROM, therefore, more studies should be conducted to confirm the conflicting findings from different countries.

Similar to our findings, Romero et al. [19] reported there was no difference in the relative abundance of vaginal microbiota between full-term and spontaneous preterm women. Study from South Korea also reported that the vaginal microbiota was similar between term and preterm samples [20]. However, Caucasian mothers with preterm birth had lower alpha diversity of than those with those full term (0.088 ± 0.082 vs 0.578 ± 0.608) [23]. In contrast, in the African American population, vaginal microbiome showed higher diversity during pregnancy in subjects who subsequently delivered to preterm birth [27]. Vaginal microbiome showed stable community richness and Shannon diversity in subjects who subsequently delivered to term, whereas vaginal richness diversity and evenness were significantly changed in pregnant women subsequently delivered to preterm birth [27]. Freida et al. also found that the association was different due to the different gestational week of sample collection. Among vaginal discharge sampled after 12 weeks of gestation, women with preterm birth had lower alpha diversity. But among those swabbed before 12 weeks of gestation, the reverse association was observed [21].

In addition to abundance and diversity, vaginal microbiota composition was also the focus of attention. The vaginal microbiome acts as a barrier to bacteria and pathogens, and the stability and dominance of *Lactobacilli* are important for reproductive health [28]. During pregnancy, the vaginal microbiome was less diverse and enriched in *Lactobacillus*, compared to vaginal microbiome in non-pregnant women [29]. A study from London reported that reduced *Lactobacillus* abundance was associated with higher risk for PPROM [26]. Another study showed that pregnant women with high abundance of *Gardnerella* and low abundance of *Lactobacillus* had higher risk for preterm birth [30]. Similar results were found in pregnant women from Peru, when vaginal discharge was sampled before 12 weeks of gestation [21]. However, among those swabbed after 12 weeks of gestation, they found that women with preterm birth had more *Lactobacillus*, [21] which was consistent with our findings. The average gestational age of sample collection was 26 gestational weeks in our study. However, there were some studies reported a weak or no association between communities dominated by *Lactobacillus* and PTB [19, 22, 23]. Stout et al. [27] also consider that neither the abundance of *Lactobacillus* nor the other rare taxa, such as Gardnerella, were significant markers of preterm birth alone. At present, absence or abundance of a specific taxa alone might not be sufficient to predict subsequent PROM or preterm birth. The findings need to be further confirmed.

There were some strengths in this study. First, we analyzed the association of diversity and composition of vaginal microbiota and PTB and PROM, using 16S data in Chinese pregnant women, which was not reported to date. Second, although the sample size was limited, we conducted the analysis after further categorized PTB into SPB and PPROM and found the similar trend. However, several limitations should be mentioned. First, we only tested the vaginal flora once during pregnancy, which might lead to different association due to sampling timing. Second, in our analysis, we did not consider the confounding of history of PTB and PROM, diet and lifestyle, because of the limited medical records. But we have considered some important pregnancy complications and basic characteristics and controlled through matching. Third, in this study, we included 21 spontaneous preterm birth and 27 indicated preterm birth (C-Section PTB cases). However, when we only kept spontaneous preterm birth through excluding C-Section PTB cases, we found the similarity results for alpha diversity although it was not statistically significant (*P*_among three groups_ = 0.0637; *P*_for the comparison of Term and TPROM_ = 0.0896). Considering the sample size, we kept all preterm birth in main results. Fourth, due to the limited sample size, we did not matched SPB with PPROM, which could lead some confounding in our analysis. Larger sample size and more intensive sample collections should be considered in the future studies.

## Conclusions

In conclusion, the alpha diversity in TPROM group was significantly lower than that in both PTB and full term group. However, there was no difference between PTB and full term. *Lactobacillus* was the most abundant in preterm birth group. Vaginal microbiota diversity and *Lactobacillus* might play an important role in the predictive of PROM and PTB. It provides new ideas for future clinical intervention related research.

## Supplementary Information

**Additional file 1:** **Supplementary Table1.** Comparisons of characteristic between YBC and those with vaginal sample.

**Additional file 2:** **Supplementary Figure 1.** The rarefaction curve of samples(a) or groups (b).

**Additional file 3:** **Supplementary Figure 2.** The comparison of alpha diversity (Chao 1 index) between groups. a. The comparison of alpha diversity (Chao 1 index) among SPB, Term and TPROM; b. The comparison of alpha diversity (Chao 1 index) among PPROM, Term and TPROM; c. The comparison of alpha diversity (Chao 1 index) between PPROM and SPB.

**Additional file 4:** **Supplementary Figure 3.** The principal coordinates analysis and the analysis of similarities among groups. a.The principal coordinates analysis among SPB, Term and TPROM groups; b. The analysis of similarities among SPB, Term and TPROM groups; c. The principal coordinates analysis among PPROM, Term and TPROM groups; d. The analysis of similarities among PPROM, Term and TPROM groups; e. The principal coordinates analysis among PPROM, Term and SPB groups; f. The analysis of similarities between PPROM and SPB.

## Data Availability

The datasets used and/or analyzed during the current study are available from the corresponding author on reasonable request.

## Abbreviations

PTB: Preterm birth
PROM: Prelabor rupture of membranes
TPROM: Term prelabor rupture of membranes
PPROM: Preterm prelabor rupture of membranes
SPB: Spontaneous preterm birth (preterm birth without PROM)
YBC: Yiwu Birth Cohort
LMP: Last menstrual period
ASV: Amplicon sequence variant
PCoA: Principal Coordinate Analysis
OTU: Operational taxonomic units
