# Supplementary material for: Vaginal flora during pregnancy and subsequent risk of preterm birth or prelabor rupture of membranes: a nested case–control study from China
Source: BMC Pregnancy Childbirth. 2023 Apr 12;23:244. doi: 10.1186/s12884-023-05564-y (PMC10091657; doi:10.1186/s12884-023-05564-y)
Supplement: Supplementary file 1 — Additional file 1: Supplementary Table1. Comparisons of characteristic between YBC and those with vaginal sample. [file 12884_2023_5564_MOESM1_ESM.docx]

| Supplementary table1. Comparisons of characteristic between YBC and those with vaginal sample. | | | |
| --- | --- | --- | --- |
| Variables | YBC (N=2980) | Those with vaginal sample (N=848) | *P* |
| Maternal age, years | 27.72 ± 4.36 | 27.63 ± 4.53 | 0.594 |
| Parity* |  |  | 0.573 |
| 0 | 1385 (50.8) | 382 (49.6) |  |
| ≥1 | 1339 (49.2) | 388 (50.4) |  |
| missing | 256 | 78 |  |
