# Supplementary figures and images for: Vaginal flora during pregnancy and subsequent risk of preterm birth or prelabor rupture of membranes: a nested case–control study from China
Source: BMC Pregnancy Childbirth. 2023 Apr 12;23:244. doi: 10.1186/s12884-023-05564-y (PMC10091657; doi:10.1186/s12884-023-05564-y)

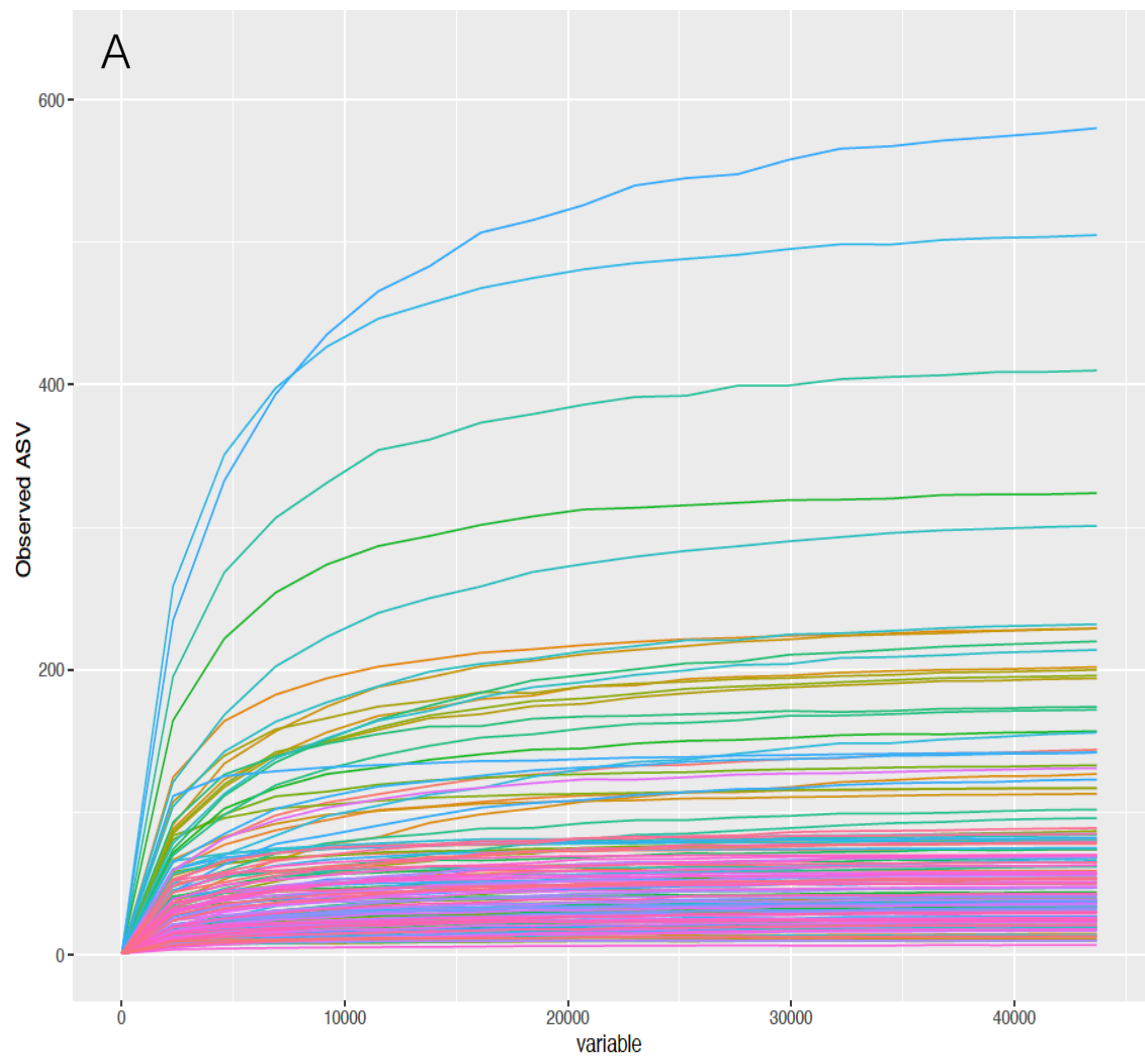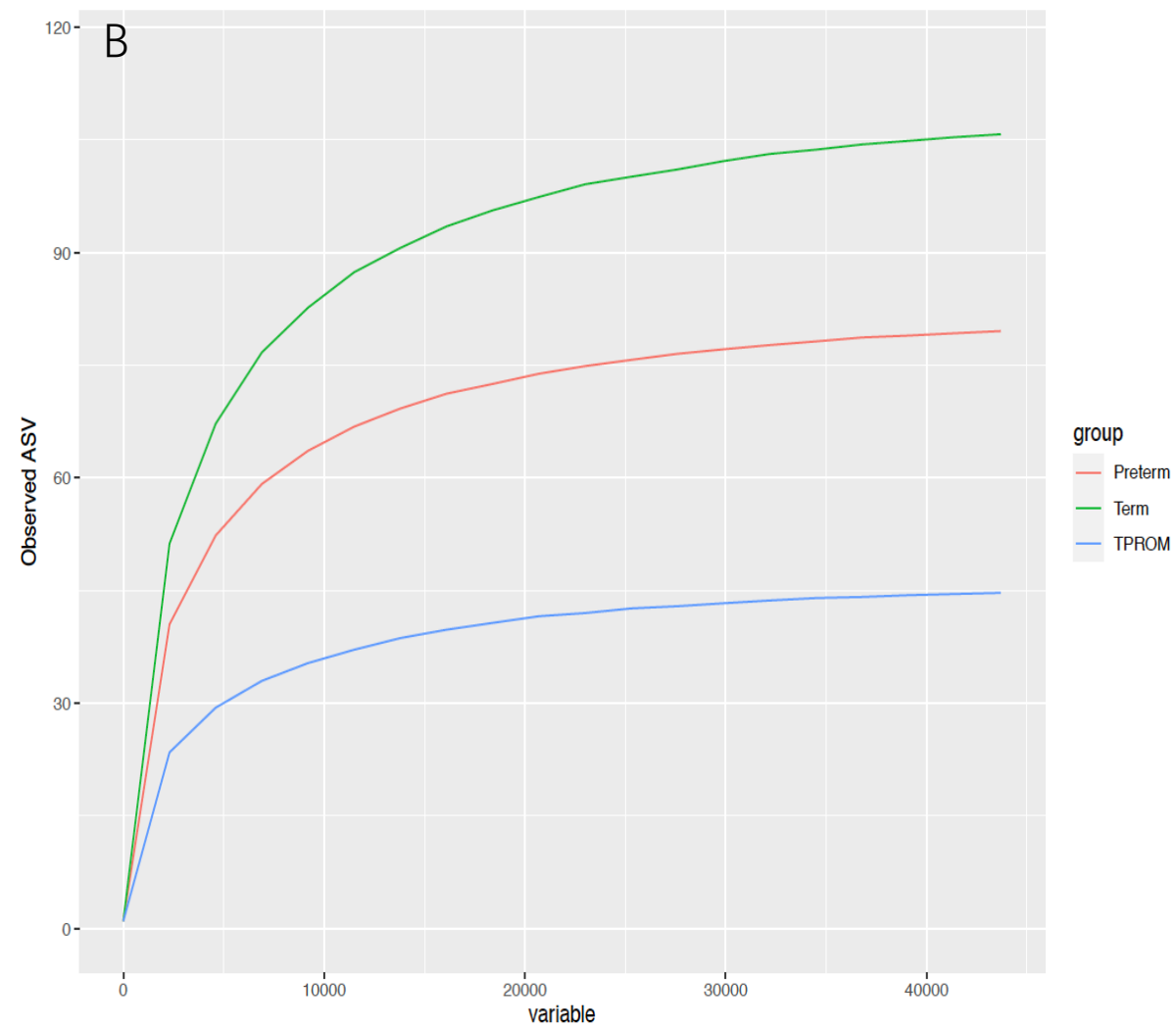

Supplement: Supplementary file 2 — Additional file 2: Supplementary Figure 1. The rarefaction curve of samples(a) or groups (b). [file 12884_2023_5564_MOESM2_ESM.pdf]

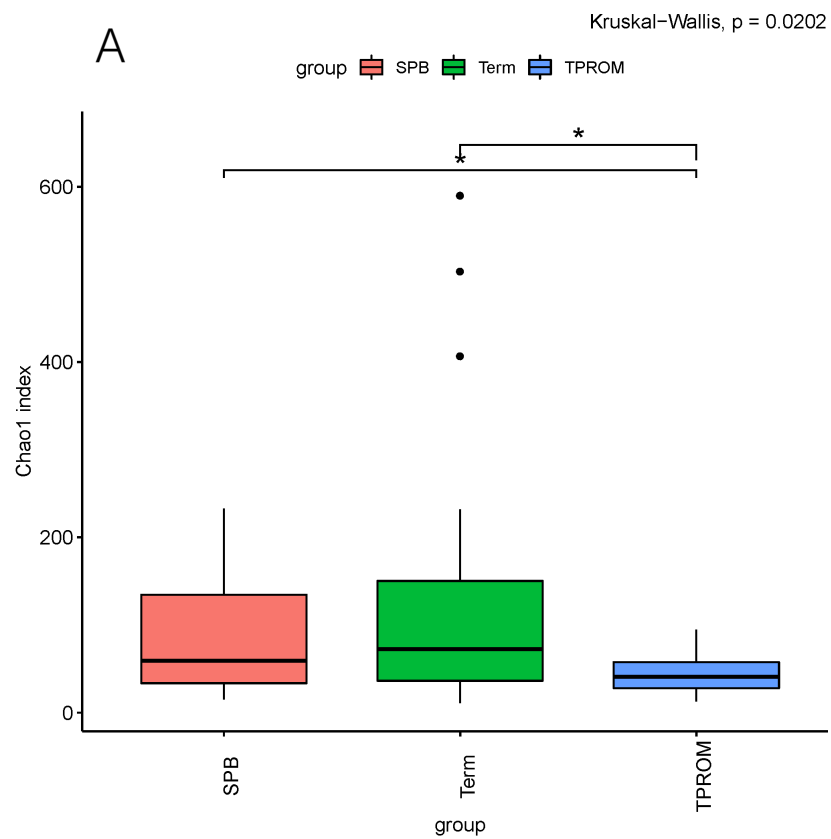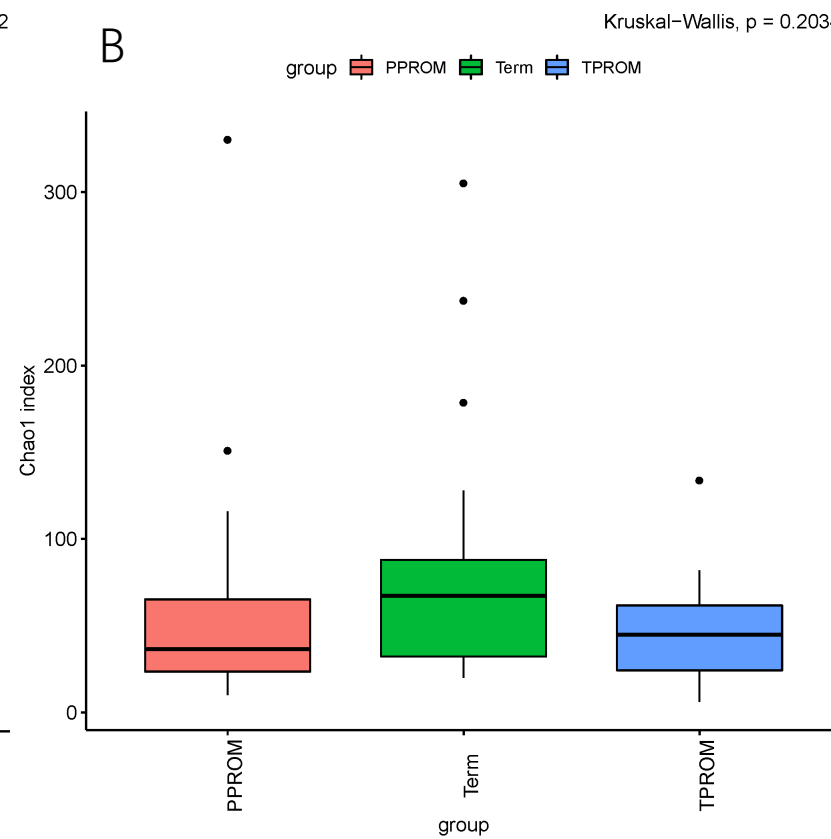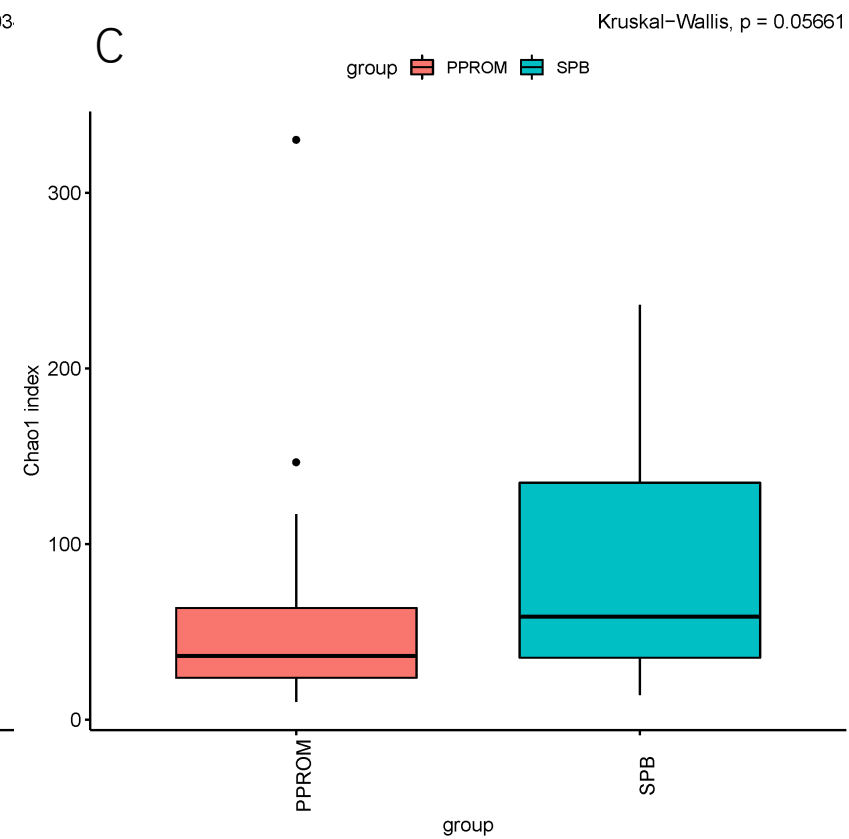

Supplement: Supplementary file 3 — Additional file 3: Supplementary Figure 2. The comparison of alpha diversity (Chao 1 index) between groups. a. The comparison of alpha diversity (Chao 1 index) among SPB, Term and TPROM; b. The comparison of alpha diversity (Chao 1 index) among PPROM, Term and TPROM; c. The comparison of alpha diversity (Chao 1 index) between PPROM and SPB. [file 12884_2023_5564_MOESM3_ESM.pdf]

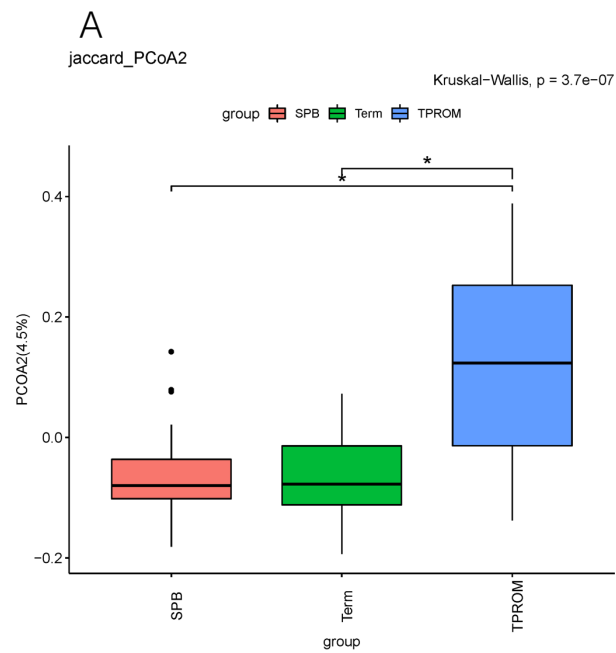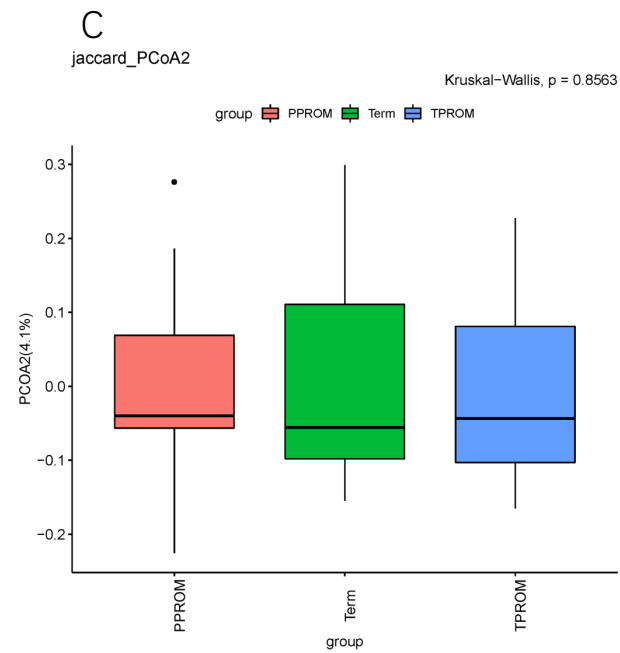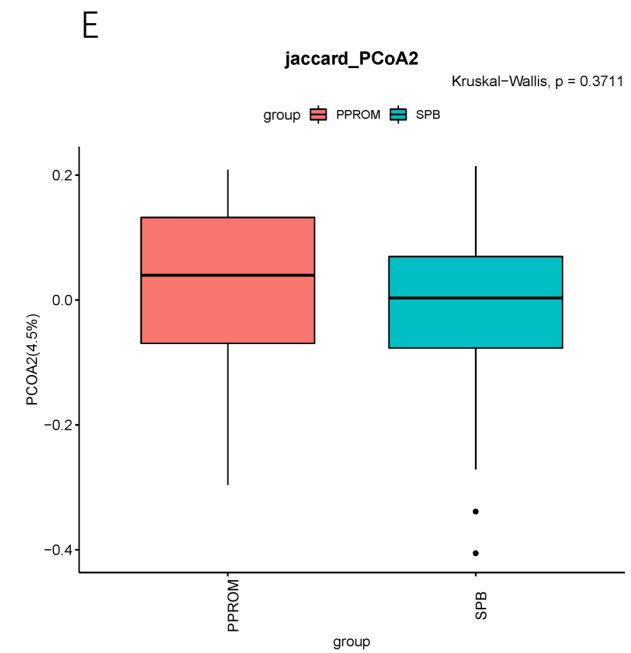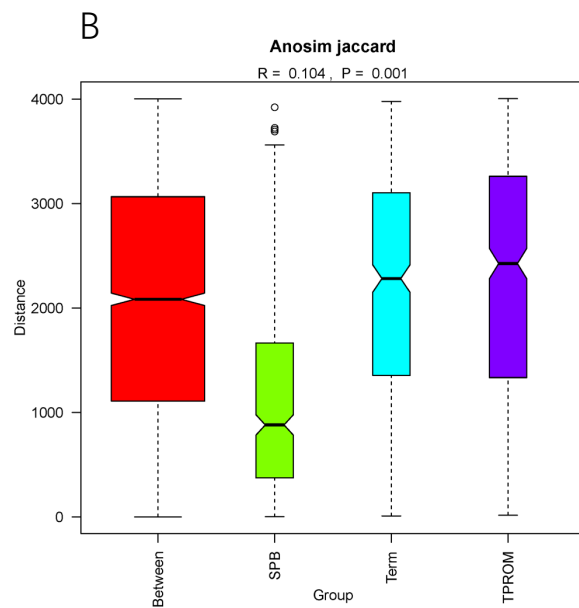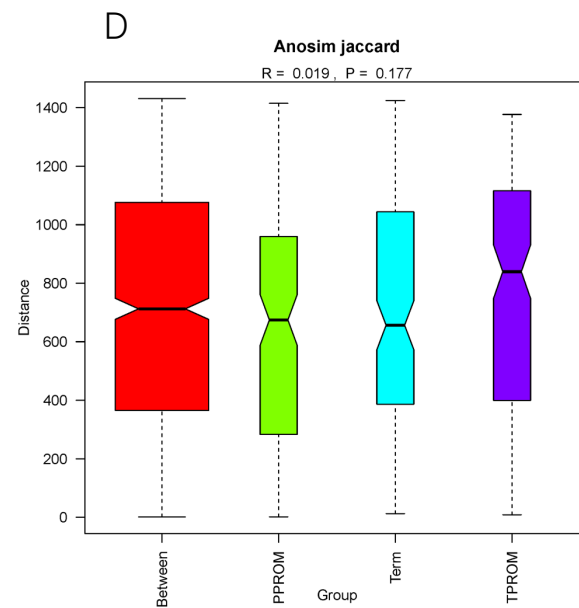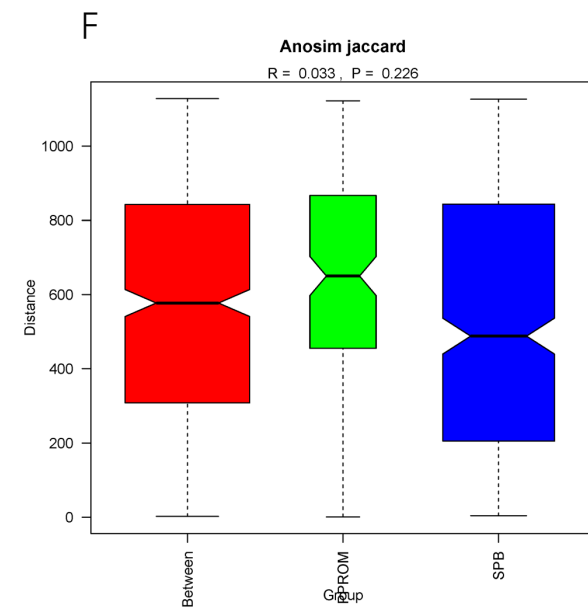

Supplement: Supplementary file 4 — Additional file 4: Supplementary Figure 3. The principal coordinates analysis and the analysis of similarities among groups. a.The principal coordinates analysis among SPB, Term and TPROM groups; b. The analysis of similarities among SPB, Term and TPROM groups; c. The principal coordinates analysis among PPROM, Term and TPROM groups; d. The analysis of similarities among PPROM, Term and TPROM groups; e. The principal coordinates analysis among PPROM, Term and SPB groups; f. The analysis of similarities between PPROM and SPB. [file 12884_2023_5564_MOESM4_ESM.pdf]
